# Supplementary material for: MYPT1-PP1β phosphatase negatively regulates both chromatin landscape and co-activator recruitment for beige adipogenesis
Source: Nat Commun. 2022 Sep 29;13:5715. doi: 10.1038/s41467-022-33363-0 (PMC9523048; doi:10.1038/s41467-022-33363-0)
Supplement: Supplementary file 1 — Supplementary Information [file 41467_2022_33363_MOESM1_ESM.pdf]

Supplementary information

MYPT1-PP1β phosphatase negatively regulates both chromatin landscape and co-activator recruitment for beige adipogenesis

Hiroki Takahashi [1, 2, 12], Ge Yang [1, 12], Takeshi Yoneshiro [2], Yohei Abe [2], Ryo Ito [1], Chaoran Yang [1], Junna Nakazono [3], Mayumi Okamoto-Katsuyama [2], Aoi Uchida [2], Makoto Arai [1], Hitomi Jin [1], Hyunmi Choi [1], Myagmar Tumenjargal [1], Shiyu Xie [1], Ji Zhang [1], Hina Sagae [1], Yanan Zhao[1], Rei Yamaguchi [1], Yu Nomura [1], Yuichi Shimizu[1], Kaito Yamada [1, 4], Satoshi Yasuda [4], Hiroshi Kimura [5], Toshiya Tanaka [6], Youichiro Wada [7], Tatsuhiko Kodama [6], Hiroyuki Aburatani [8], Min-Sheng Zhu [9], Takeshi Inagaki [2, 10], Timothy F. Osborne [11], Takeshi Kawamura [7], Yasushi Ishihama [3], Yoshihiro Matsumura [1, 2, *], Juro Sakai [1, 2, *]

[1] Division of Molecular Physiology and Metabolism, Tohoku University Graduate School of Medicine, Sendai 980-8574, Japan

[2] Division of Metabolic Medicine, Research Center for Advanced Science and Technology, The University of Tokyo, Tokyo 153-8904, Japan

[3] Department of Molecular and Cellular BioAnalysis, Graduate School of Pharmaceutical Sciences, Kyoto University, Kyoto 606-8501, Japan

[4] Department of Cardiovascular Medicine, Tohoku University Graduate School of Medicine, Sendai 980-8574, Japan

[5] Cell Biology Unit, Institute of Innovative Research, Tokyo Institute of Technology, Yokohama 226-8503, Japan

[6] Department of Nuclear Receptor Medicine, Laboratories for Systems Biology and Medicine, Research Center for Advanced Science and Technology, The University of Tokyo, Tokyo 153-8904, Japan

[7] Isotope Science Center, The University of Tokyo, Tokyo 113-0032, Japan

[8] Genome Science and Medicine Division, Research Center for Advanced Science and Technology, The University of Tokyo, Tokyo 153-8904, Japan

[9] Model Animal Research Center and Ministry of Education (MOE) Key Laboratory of Model Animal for Disease Study, Nanjing University, Nanjing 210061, China

[10] Laboratory of Epigenetics and Metabolism, Institute for Molecular and Cellular Regulation, Gunma University, Gunma 371-8512, Japan

[11] Institute for Fundamental Biomedical Research, Johns Hopkins All Children’s Hospital,

and Medicine in the Division of Endocrinology, Diabetes and Metabolism of the Johns Hopkins University School of Medicine, 600 Fifth Street S. St. Petersburg, FL 33701, USA
[12] These authors contributed equally
[*] Correspondence should be addressed to Y.M. or J.S. (e-mail: matsumura-y@lsbm.org or jmsakai@med.tohoku.ac.jp)

Supplementary Tables

Supplementary Table 1. List of genes upregulated by MYPT1 depletion in Fig. 1g.

RNA-seq MA plot of 3,356 beige-selective genes (FC of RPKM (si-Ctrl day 8/day 0 > 1.5), RPKM > 1) in primary scWAT cultures of WT mice transfected with si-Ctrl or si-*Mypt1* #2 and differentiated for beige adipocyte. Upregulated (FC > 1.5) beige-selective genes following MYPT1 depletion are shown.

Fabp3-ps1	*Elovl3*	*Gsta4*	*Iglon5*	*Aif1l*
Clec3b	*Ly6a*	*Hist2h2aa2*	*Chst13*	*Ppp2r5b*
Ucp1	*AL772271.1*	*Hephl1*	*Plin5*	*Krt79*
Fabp3	*Mir126*	*Chst1*	*Ace*	*Hcn2*
Itgad	*Orm1*	*Gm2308*	*Acyp2*	*Islr*
Hist1h4b	*Aldh3b2*	*Atp2a3*	*Arc*	*Gm16641*
Ly6c1	*Acp5*	*Chkb*	*Iigp1*	*1810034E14Rik*
Hist1h4c	*Tuba8*	*mt-Te*	*Syngr1*	*Acacb*
Cidea	*Gnao1*	*A830052D11Rik*	*Krt36*	*C1rb*
Cyp4b1	*1110059M19Rik*	*Cox6b2*	*Slc25a42*	*Hist1h2bg*
Ubd	*Pltp*	*Gm15317*	*Hist1h2be*	*Mmp15*
Cox8b	*Gm4951*	*Mrps36-ps2*	*Adck3*	*Apol6*
Hist1h4h	*1700056E22Rik*	*Gm7251*	*Sparcl1*	*Fam195a*
Tnxb	*Spon1*	*Tmem45b*	*AC154646.1*	*Gm5514*
Otop1	*Gm6293*	*Rbpms2*	*Syt12*	*Hdhd3*
Slc6a13	*Lctl*	*Pank1*	*Ptger3*	*Slc7a10*
Impa2	*Gm12347*	*Gm6181*	*Aatk*	*Hadha*
Sncg	*Acaa2*	*Odf3l1*	*Nnat*	*Letmd1*
Car4	*Cpt1b*	*Gbp10*	*Adh1*	*Ehhadh*
Sema3b	*Tlr5*	*Gm16559*	*Gm17147*	*Epas1*
Ly6i	*Hist2h2aa1*	*Apoc3*	*Sprr1a*	*Dhrs4*
Lipg	*Lrrc29*	*Adamts15*	*Poln*	*Gm3222*
Aadac	*Cdkn1c*	*SNORA5*	*Dusp8*	*Gm17168*

Supplementary Table 2. List of reagents and resources.

Antibodies			
Reagent or Resource	Source	Identifier	Dilution or concentration
Anti-mouse JMJD1A	Our laboratory	IgG-F0618	10 μg/mL for IB<br>8.3 μg/mL for IP<br>25 μg/mL for ChIP
Anti-mouse JMJD1A	Our laboratory	IgG-F0231	4 μg/mL for IB<br>12 μg/mL for IP<br>25 μg/mL for ChIP
Anti-human JMJD1A	Our laboratory	IgG-F3640	10 μg/mL for IP
Anti-P-JMJD1A (pSer265)	Our laboratory	11890-2	1:500 for IB
Anti-RLC	Cell Signaling Technology	Cat#8505	1:500 for IB
Anti-P-RLC (pSer19)	Cell Signaling Technology	Cat #3671	1:500 for IB
Anti-MYPT1	Cell Signaling Technology	Cat #2634	1:500 for IB
Anti-P-MYPT1 (pThr696)	Sigma-Aldrich	Cat #ABS45	1:500 for IB
Anti-ACTB	Sigma-Aldrich	Cat #A5441	1:5,000 for IB
Anti-V5	Invitrogen	Cat #R960-25	1 μg/mL for IB
Anti-H3K9me2	Dr. Hiroshi Kimura, Tokyo Institute of Technology	IgG-6D11	2 μg/mL for ChIP
Anti-UCP1	Abcam	Cat #ab23841	1:5000 for IHC
Anti-mCherry	Abcam	Cat #ab125096	1:150 for IHC
Anti-UCP1	R&D Systems	Cat #MAB6158	0.5 μg/mL for IB
Anti-TOM20	Proteintech	Cat #11802-1-AP	0.8 μg/mL for IB
Anti-FLAG	Sigma-Aldrich	Cat#F3165	1 μg/ml for IP
Anti-mouse IgG-HRP	Sigma-Aldrich	Cat#A4416	1:10,000 for IB
Anti-rabbit IgG-HRP	Sigma-Aldrich	Cat#A0545	1:10,000 for IB

Bacterial plasmid and Virus Vector		
Reagent or Resource	Source	Identifier
pMXs-IRES-Puro	Cosmo Bio	Cat#RTV-014
pMXs-IRES-Zeo	Ref [1]	

pAAV-CMV-mCherry	Dr. Akihiro Yamanaka, Nagoya University	
pAAV-CMV-mCherry-2A-Cre	Dr. Akihiro Yamanaka, Nagoya University	
pHelper	Agilent	Cat#240071
pAAV-RC8	Dr. James M Wilson, The University of Pennsylvania	
AxCANLacZ	RIKEN BRC	Cat#RDB01749
AxCANCre	RIKEN BRC	Cat#RDB01748
Chemicals, Peptides, and Recombinant Proteins		
Reagent or Resource	Source	Identifier
DMEM High Glucose	Sigma-Aldrich	Cat#D6429
Fetal Bovine Serum	Thermo Fisher Scientific	Cat#10270
Penicillin-Streptomycin Mixed Solution	Nacalai Tesque	Cat#09367-34
3-Isobutyl-1-methylxanthine	Sigma-Aldrich	Cat#I5879
Dexamethasone	Sigma-Aldrich	Cat#D4902
Insulin	Sigma-Aldrich	Cat#I5523
Rosiglitazone	FUJIFILM Wako	Cat#184-02651
Isoproterenol	Sigma-Aldrich	Cat#I6504
H89	Cell Signaling Technology	Cat#9844
Blebbistatin	Sigma-Aldrich	Cat#203389
Norepinephrine	Sigma-Aldrich	Cat#A9512
Puromycin	Sigma-Aldrich	Cat#P8833
Zeocin	InvivoGen	Cat#ant-zn-1p
G418	Sigma-Aldrich	Cat#A1720
Oil Red O	Nacalai Tesque	Cat#25633-92
Super Signal West Dura Extended Duration Substrate	Thermo Fisher Scientific	Cat#34075
Lipofectamine RNAiMAX Transfection Reagent	Thermo Fisher Scientific	Cat#13778150

Stealth RNAi siRNA Negative Control Med GC Duplex #2	Thermo Fisher Scientific	Cat#12935-112
ON-TARGETplus Non-targeting Control Pool	Horizon Discovery	Cat#D-001810-10-20
KOD Plus Neo DNA polymerase	Toyobo	Cat#KOD-401
ISOGEN Reagent	Nippon Gene	Cat#315-02504
Formaldehyde Solution	FUJIFILM Wako	Cat#064-00406
Sequencing Grade Modified Trypsin	Promega	Cat#V5111
Ethylene glycol bis(succinimidylsuccinate)	Thermo Fisher Scientific	Cat#21565
Dynabeads Protein G for Immunoprecipitation	Thermo Fisher Scientific	Cat#10004D
Protein G Sepharose 4 Fast Flow	GE Healthcare	Cat#17061801
Critical Commercial Assays		
Reagent or Resource	Source	Identifier
QIAquick PCR Purification Kit	Qiagen	Cat#28106
Qubit Double-stranded DNA High Sensitivity Assay Kit	Thermo Fisher Scientific	Cat#32854
TruSeq ChIP Library Preparation Kit	Illumina	Cat#RS-122-2001 or RS-122-2002
KAPA Hyper Prep Kit	Kapa Biosystems	Cat#KK8502
Deposited Data		
Reagent or Resource	Source	Identifier
RAW and analyzed RNA-seq data	This paper	GSE202506
RAW and analyzed ChIP-seq data	This paper	GSE202506
Phosphoproteomics data	This paper	PXD031210, PXD031896, PXD031897

Experimental Model: Cell Line		
Reagent or Resource	Source	Identifier
3T3-L1	ATCC	Cat#ATCC CL-173
Plat-E	Cosmo Bio	Cat#RV-101
NIH-3T3	ATCC	Cat#ATCC CRL-1658
AAV293	Agilent	Cat#240073

Software and Algorithms		
Reagent or Resource	Source	Identifier
HOMER4.11	Ref [2]	http://homer.ucsd.edu/homer/
Bowtie2 2.4.2	Ref [3]	http://bowtie-bio.sourceforge.net/bowtie2/index.shtml
Trimmomatic0.39	Ref [4]	http://www.usadellab.org/cms/?page=trimmomatic
Samtools1.12	Ref [5]	http://www.htslib.org/
Bedtools2.30.0	Ref [6]	https://bedtools.readthedocs.io/en/latest/
STAR 2.7.9a	Ref [7]	http://code.google.com/p/rna-star/.
GFOLD1.1.4	Ref [8]	https://zhanglab.tongji.edu.cn/softwares/GFOLD/index.html
FASTP0.20.1	Ref [9]	https://github.com/OpenGene/fastp
Deeptools3.5.1	Ref [10]	https://deeptools.readthedocs.io/en/develop/

Supplementary Table 3. List of RT-qPCR primers.

Gene	Forward primer	Reverse primer
Ppib	5'-GGAGATGGCACAGGAGGAA-3'	5'-GCCCGTAGTGCTTCAGCTT-3'
Ucp1	5'-AAGCTGTGCGATGTCCATGT-3'	5'-AAGCCACAAACCCTTTGAAAA-3'
Cidea	5'-GGTTCAAGGCCGTGTTAAGG-3'	5'-CGTCATCTGTGCAGCATAGG-3'
Pgc1a	5'-AACCACACCCACAGGATCAGA-3'	5'-TCTTCGCTTTATTGCTCCATGA-3'
Prdm16	5'-GCACGGTGAAGCCATTCATATG-3	5'-TCGGCGTGCATCCGCTTGTG-3'
Dio2	5'-GTCCGCAAATGACCCCTTT-3'	5'-CCCACCCACTCTCTGACTTTC-3'
Cpt1b	5'-GCTGCCGTGGGACATTC-3'	5'-CTTGGCTACTTGGTACGAGTTCTC-3'
Pparg	5'-CAAGAATACCAAAGTGCGATCAA-3'	5'-GAGCTGGGTCTTTTCAGAATAATAAG-3'
Cox8b	5'-CACTTCCGCCGTGGAGC-3'	5'-GTGGGCTAAGACCCATCCTG-3'
Pgc1b	5'-GAGGGCTCCGGCACTTC-3'	5'-CGTACTTGCTTTTCCCAGATGA-3'
Ppara	5'-ACAAGGCCTCAGGGTACCA-3'	5'-GCCGAAAGAAGCCCTTACAG-3'
Elovl3	5'-TTCTCACGCGGGTTAAAAATG-3'	5'-GGGCCTTAAGTCCTGAAACGT-3'
Fabp4	5'-AGTGAAAACTTCGATGATTACATGAA-3'	5'-GCCTGCCACTTTCCTTGTG-3'
Tfam	5'-CCGAAGTGTTTTTCCAGCAT-3'	5'-GGCTGCAATTTTCCTAACCA-3'
Otop1	5'-TACATGCAGCGCCGCCTCAT-3'	5'-TCAAGCATCCCAGGACGACA-3'
Bmp8b	5'-CACTTCCGCCGTGGAGC-3'	5'-GTGGGCTAAGACCCATCCTG-3'
Adipoq	5'-CAGTGGATCTGACGACACCAA-3'	5'-GAACAGGAGAGCTTGCAACAGT-3'
Tmem26	5'-ACCCTGTCATCCCACAGAG-3'	5'-TGTTTGGTGGAGTCCTAAGGTC-3'
Cd137	5'-CGTGCAGAACTCCTGTGATAAC-3'	5'-GTCCACCTATGCTGGAGAAGG-3'
Cited1	5'-ATTTATCGGACTTCTGCCCAG-3'	5'-TTGCGATCCTTCACTCCAAG-3'
Tbx1	5'-GGCAGGCAGACGAATGTTC-3'	5'-TTGTCATCTACGGGCACAAAG-3'
Shox2	5'-CCCACTATCCAGACGCTTTC-3'	5'-ATAGGGTGCAACTCTACAAGC -3'
Ppp1ca	5'-GACCCTCATGTGTTCCTTCCA-3'	5'-CCGCTGAACTGCCCATACTT-3'
Ppp1cb	5'-GTCACCAGACCTACAATCTATGGAA-3'	5'-AAGTCACAAAGCAAACCTGTATCAG-3'
Ppp1cc	5'-GCTGTCATGGAGGTTTATCAC-3'	5'-GATCTGGTACATCAGTTGGTC-3'
Rlc + RLC	5'-CACCAAGAAGCGCCCTCAG-3'	5'-TGTACAGCTCATCCACTTCCTC-3'
Taz	5'-GAAGGTGATGAATCAGCCTCTG-3'	5'-GTTCTGAGTCGGGTGGTTCTG-3'
Ctgf	5'-TTGACAGGCTTGGCGATT-3'	5'-GTTACCAATGACAATACCTTCTGC-3'
Cyr61	5'-GTGAAGTGCGTCCTTGTGGACA-3'	5'-CTTGACACTGGAGCATCCTGCA-3'
Actb	5'-CCGTGAAAAGATGACCCAGATC-3'	5'-CACAGCCTGGATGGCTACGT-3'
Pdgfra	5'-TCATCCCCCTGCCAGACATT-3'	5'-GATGGCACTCTCTTCCGAAGTC-3'
mCherry	5'-GAACGGCCACGAGTTCGAGA-3'	5'-CTTGGAGCCGTACATGAACTGAGG-3'
Mylk	5'-TCATCCTTGAACCAGACCAC-3'	5'-CCTTATTTCTCTAAGACCATCCGT-3'
Mypt1	5'-AGAGCTCAAAATGTTACCAGACTT-3'	5'-AGTCCTGCTGCTTTGCTTCT-3'

Supplementary Table 4. List of ChIP-qPCR primers.

Gene	Forward Primer	Reverse Primer
Actb (Intron 1)	5'-TGAGGTACTAGCCACGAGAGAG-3'	5'-ACACCCGCCACCAGGTAAGCA-3'
Ucp1 (-13 kb)	5'-GCAACCCTCTCCCATCAGTG-3'	5'-GCCTAACACCGTGCTTCTCA-3'
Ucp1 (-4.8 kb)	5'-TGCAACCCCTCACCTTTTAC-3'	5'-CTCCTTCCATCATCCCTTCA-3'
Ucp1 (-2.5 kb)	5'-TCACCCTTGACCACACTGAA-3'	5'-GTGAGGCTGATATCCCCAGA-3'
Ucp1 (TSS)	5'-TGCCAAGTCCCACTAGCAG-3'	5'-ACCCGTTAAGCCCAGATTG-3'
Ppara (-10 kb)	5'-TGGCCGGGAGGAACTG-3’	5'-GGCAGGGACAATCTCTTTGTG-3'
Cidea (-13.5 kb)	5'-CACCGCTTCACTTTGTCCTTT-3'	5'-GAGCACCCGGTTTGACAGT-3'
Cidea (TSS)	5'-CACGCACACCTGCTTCTCTA-3'	5'-GATGTTGGTGGCTCTTGTCA-3'

Supplementary Table 5. Details of age and sex of mice.

Mouse Strain	Line of *Mypt1* floxed mice	Sex	Age	Figure
C57BL/6J		Male	16-18	Supplementary Figure 5b, 5c
		Male	7	Supplementary Figure 5d (left)
Mypt1$^{flox/flox}$	Line 2	Female	21-34	Figure 5b, f
		Male	18	Figure 5c, 5d, 5e, Supplementary Figure 5d (right), 5e
Mypt1$^{+/flox}$::*Pdgfra*-Cre		Female	18	Figure 5g (left, right)
		Female	18-19	Figure 5g (inset)
		Female	9-19	Figure 5h, Supplementary Figure 5n
		Female	19	Figure 5i
		Male	5-7	Figure 5j
		Male	21-23	Figure 5k
		Male	22-23	Figure 5l, Supplementary Figure 5r
		Female	33	Supplementary Figure 5f
		Female	9	Supplementary Figure 5o, 5p
		Male	6-7	Supplementary Figure 5q
Mypt1$^{flox/flox}$::*Adipoq*-Cre	Line 1	Male	8	Supplementary Figure 5j, 5k, 5l

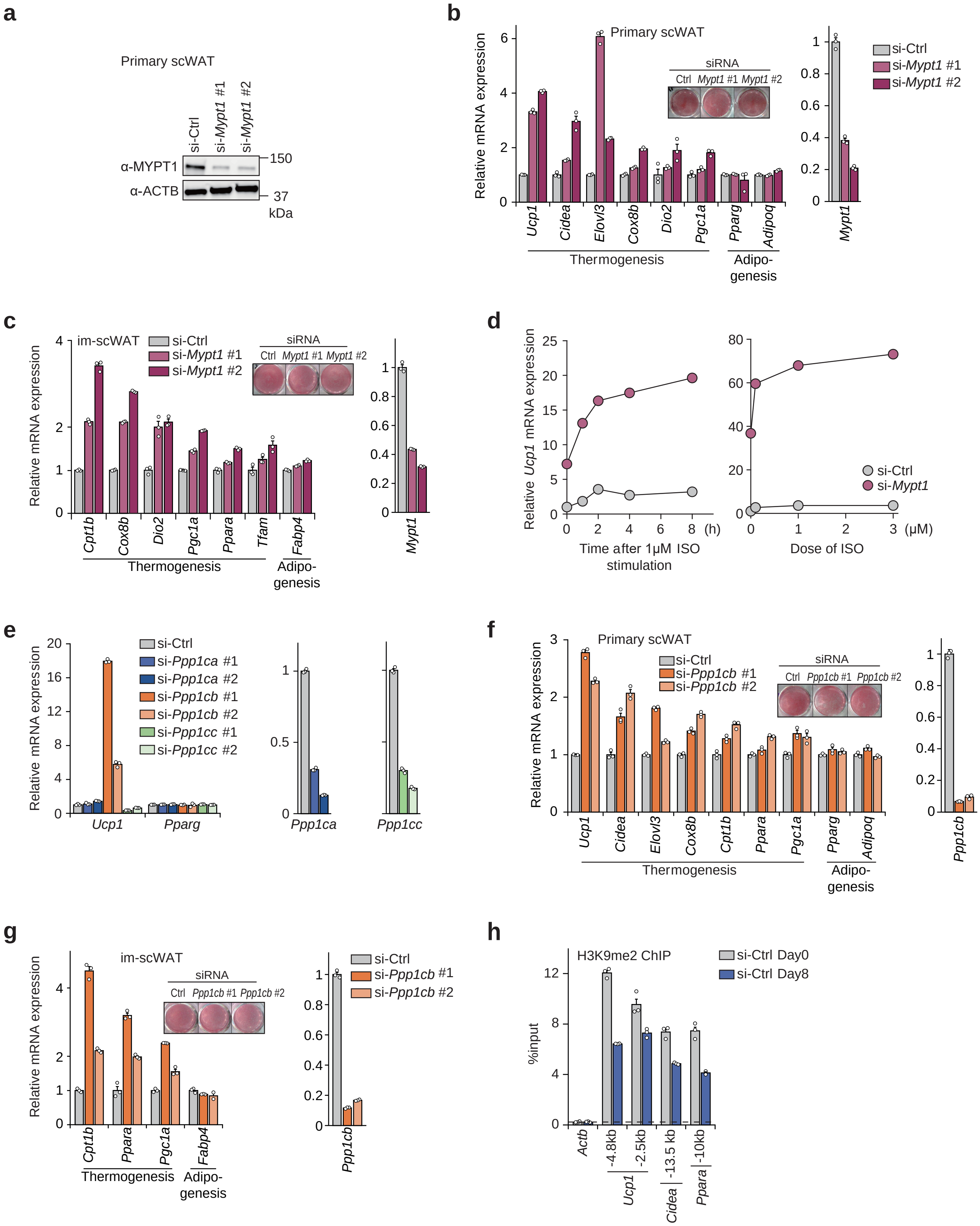
a
Primary scWAT
si-Ctrl
si-Mypt1 #1
si-Mypt1 #2
α-MYPT1
α-ACTB
150
37
kDa
b
Primary scWAT
Relative mRNA expression
siRNA
Ctrl Mypt1 #1 Mypt1 #2
si-Ctrl
si-Mypt1 #1
si-Mypt1 #2
Ucp1
Cidea
Elovl3
Cox8b
Dio2
Pgc1a
Pparg
Adipoq
Mypt1
Thermogenesis
Adipo-genesis
c
im-scWAT
si-Ctrl
si-Mypt1 #1
si-Mypt1 #2
siRNA
Ctrl Mypt1 #1 Mypt1 #2
Relative mRNA expression
Cpt1b
Cox8b
Dio2
Pgc1a
Ppara
Tfam
Fabp4
Mypt1
Thermogenesis
Adipo-genesis
d
Relative Ucp1 mRNA expression
Time after 1μM ISO stimulation
(h)
Dose of ISO
(μM)
si-Ctrl
si-Mypt1
e
Relative mRNA expression
si-Ctrl
si-Ppp1ca #1
si-Ppp1ca #2
si-Ppp1cb #1
si-Ppp1cb #2
si-Ppp1cc #1
si-Ppp1cc #2
Ucp1
Pparg
Ppp1ca
Ppp1cc
f
Primary scWAT
Relative mRNA expression
si-Ctrl
si-Ppp1cb #1
si-Ppp1cb #2
siRNA
Ctrl Ppp1cb #1 Ppp1cb #2
Ucp1
Cidea
Elovl3
Cox8b
Cpt1b
Ppara
Pgc1a
Pparg
Adipoq
Ppp1cb
Thermogenesis
Adipo-genesis
g
im-scWAT
siRNA
Ctrl Ppp1cb #1 Ppp1cb #2
Relative mRNA expression
si-Ctrl
si-Ppp1cb #1
si-Ppp1cb #2
Cpt1b
Ppara
Pgc1a
Fabp4
Ppp1cb
Thermogenesis
Adipo-genesis
h
H3K9me2 ChIP
si-Ctrl Day0
si-Ctrl Day8
%input
Actb
-4.8kb
-2.5kb
Ucp1
-13.5 kb
Cidea
-10kb
Ppara

Supplementary Fig. 1 MYPT1-PP1β dephosphorylates pS265-JMJD1A and suppresses thermogenic gene expression through the modulation of H3K9me2.

(**a**) Immunoblotting with anti-MYPT1 and anti-ACTB antibody using WCL of primary pre-adipocytes isolated from SVF of scWAT of WT mice transfected with si-Ctrl or si-*Mypt1*. (**b**) mRNA levels for thermogenic genes, general adipogenic genes (day 8), and *Mypt1* (day 0) in primary pre-adipocytes isolated from SVF of scWAT of WT mice differentiated for beige adipogenesis transfected with si-Ctrl or si-*Mypt1*. ORO staining on day 8 (inset). (**c**) Thermogenic genes, general adipogenic genes (day 8), and *Mypt1* (day 0) mRNA levels in im-scWAT transfected with si-*Mypt1*. ORO staining on day 8 (inset). (**d**) mRNA levels of *Ucp1* in day 8 im-scWAT cells transfected with si-Ctrl or si-*Mypt1* #2 after 1 μM ISO treatment at the indicated time points (left). mRNA levels of *Ucp1* in day 8 im-scWAT cells transfected with si-Ctrl or si-*Mypt1* #2 after treatment of 0, 1, 2, 3 μM ISO for 1 h (right). (**e**) mRNA levels of *Ucp1*, *Pparg* (left, day 8), *Ppp1ca* (middle, day 0), and *Ppp1cc* (right, day 0) in im-scWAT cells transfected with si-Ctrl, si-*Ppp1ca*, si-*Ppp1cb*, or si-*Ppp1cc.* (**f**) mRNA levels for thermogenic genes, general adipogenic genes (day 8), and *Ppp1cb* (day 0) in primary pre-adipocytes isolated from SVF of scWAT of WT mice differentiated for beige adipogenesis transfected with si-Ctrl or si-*Ppp1cb*. ORO staining on day 8 (inset). (**g**) Thermogenic genes, general adipogenic genes (day 8), and *Ppp1cb* (day 0) mRNA levels in im-scWAT transfected with si-*Ppp1cb* #1. ORO staining on day 8 (inset). (**h**) H3K9me2 ChIP-qPCR in im-scWAT cells differentiated for beige adipogenesis on day 0 and day 8. **a-h** Representative of three (**b**, **c**, **f**, **g**) or two (**a**, **d**, **e**, **h**) independent experiments. Data are mean ± SEM of three technical replicates in **b-h**. Source data are provided as a Source data file.

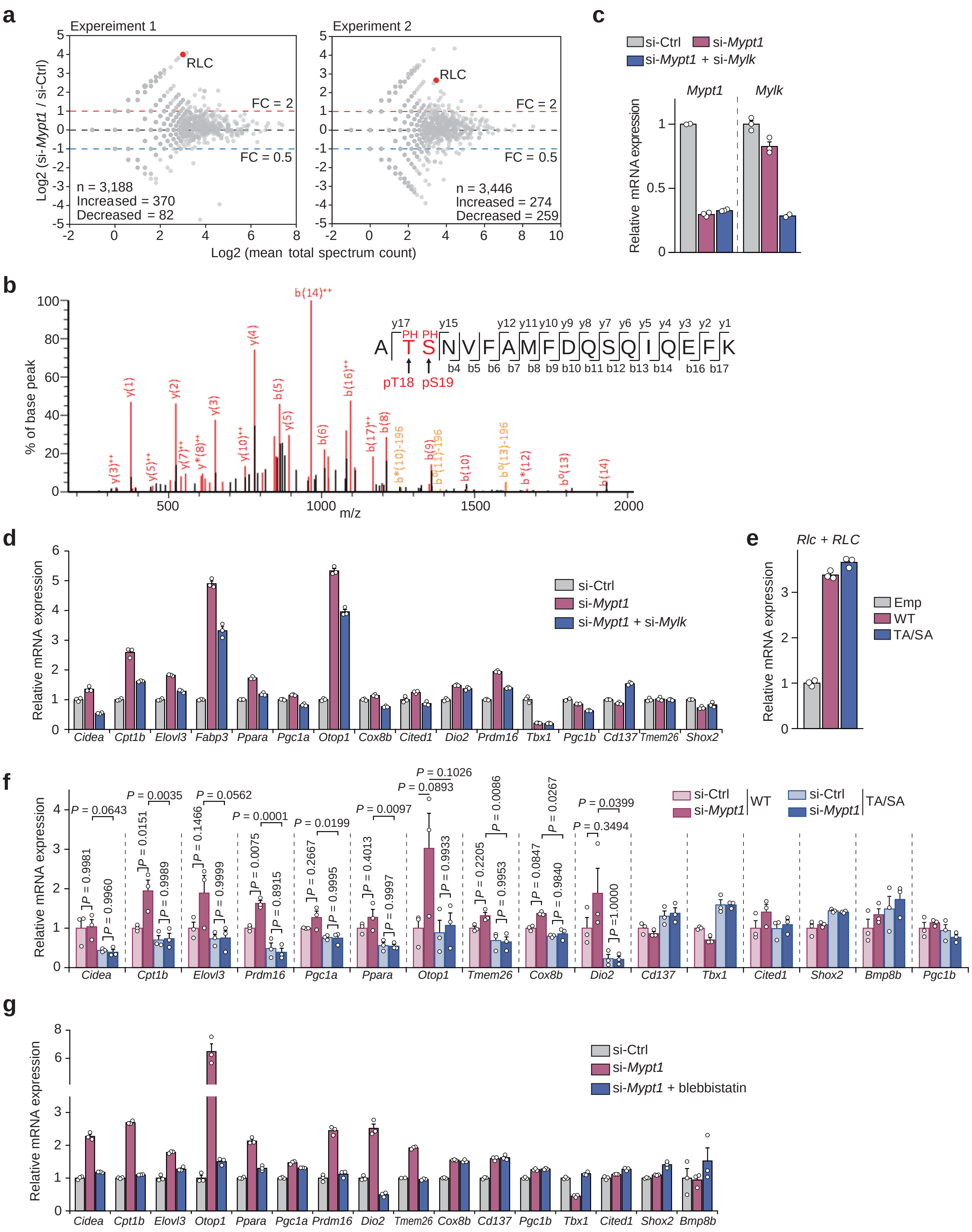
a
Expereiment 1
Experiment 2
RLC
FC = 2
FC = 0.5
n = 3,188
Increased = 370
Decreased = 82
n = 3,446
Increased = 274
Decreased = 259
Log2 (si-Mypt1 / si-Ctrl)
Log2 (mean total spectrum count)
b
% of base peak
m/z
A T S N V F A M F D Q S Q I Q E F K
pT18
pS19
c
si-Ctrl
si-Mypt1
si-Mypt1 + si-Mylk
Mypt1
Mylk
Relative mRNA expression
d
si-Ctrl
si-Mypt1
si-Mypt1 + si-Mylk
Relative mRNA expression
Cidea Cpt1b Elovl3 Fabp3 Ppara Pgc1a Otop1 Cox8b Cited1 Dio2 Prdm16 Tbx1 Pgc1b Cd137 Tmem26 Shox2
e
Rlc + RLC
Emp
WT
TA/SA
Relative mRNA expression
f
si-Ctrl
si-Mypt1
WT
TA/SA
Relative mRNA expression
Cidea Cpt1b Elovl3 Prdm16 Pgc1a Ppara Otop1 Tmem26 Cox8b Dio2 Cd137 Tbx1 Cited1 Shox2 Bmp8b Pgc1b
g
si-Ctrl
si-Mypt1
si-Mypt1 + blebbistatin
Relative mRNA expression
Cidea Cpt1b Elovl3 Otop1 Ppara Pgc1a Prdm16 Dio2 Tmem26 Cox8b Cd137 Pgc1b Tbx1 Cited1 Shox2 Bmp8b

Supplementary Fig. 2 MYPT1-PP1β regulates beige adipogenesis via RLC phosphorylation

(**a**) MA plots of si-*Mypt1* #2 / si-Ctrl phosphoproteome in im-scWAT cells on day 0 (Label-free Quantification) Left: Experiment 1, Right: Experiment 2. X-axis, log2 mean of total spectrum count:: Y-axis, log2 fold change of total spectrum count. The numbers of total phosphopeptides (n), phosphopeptides with increased (Increased: fold change > 2 and total spectrum count of si-*Mypt1* transfected cells > 3) and decreased (Decreased: fold change < 0.5 and total spectrum count of si-Ctrl transfected cells > 3) phosphorylation levels by MYPT1 depletion are shown. (**b**) Post-translational modifications of RLC identified by the phosphoproteomic analysis described in Figure 2b. Shown is a MS/MS spectrum of the P-RLC fragment from A17 to K34. (**c**) *Mypt1* and *Mylk* mRNA levels were quantified by RT-qPCR on day 0 in im-scWAT cells transfected with si-Ctrl alone, both si-*Mypt1* #2 and si-Ctrl, or both si-*Mypt1* #2 and si-*Mylk*. Data are mean ± SEM of three technical replicates. (**d**) Thermogenic gene expression in day 8 im-scWAT transfected with si-Ctrl, si-Ctrl+si-*Mypt1* #2, or si-*Mypt1* #2+si-*Mylk*. (**e**) Mouse and human RLC mRNA levels in empty vector, WT-, or T18A/S19A-human RLC-transduced im-scWAT cells on day 0. (**f**) Changes in the expression of thermogenic genes in im-scWAT adipocytes at day 8 of differentiation overexpressing WT or T18A/S19A human RLC by depletion of *Mypt1* (si-*Mypt1* #2). (**g**) Effects of thermogenic gene expression by *Mypt1* depletion and blebbistatin treatment during beige adipogenesis. Cultured im-scWATs were transfected with control siRNA (si-Ctrl) or si-*Mypt1* #2, treated with 10 μM blebbistatin, and induced for differentiation of beige adipocytes. On day 8, cells were harvested for qPCR. **c**, **d**, **e**, **g** Representative of two independent experiments. Data are mean ± SEM of three technical replicates in **c**, **d**, **e**, and **g**. **f** One-way ANOVA with Tukey's multiple comparisons test. Source data are provided as a Source data file.

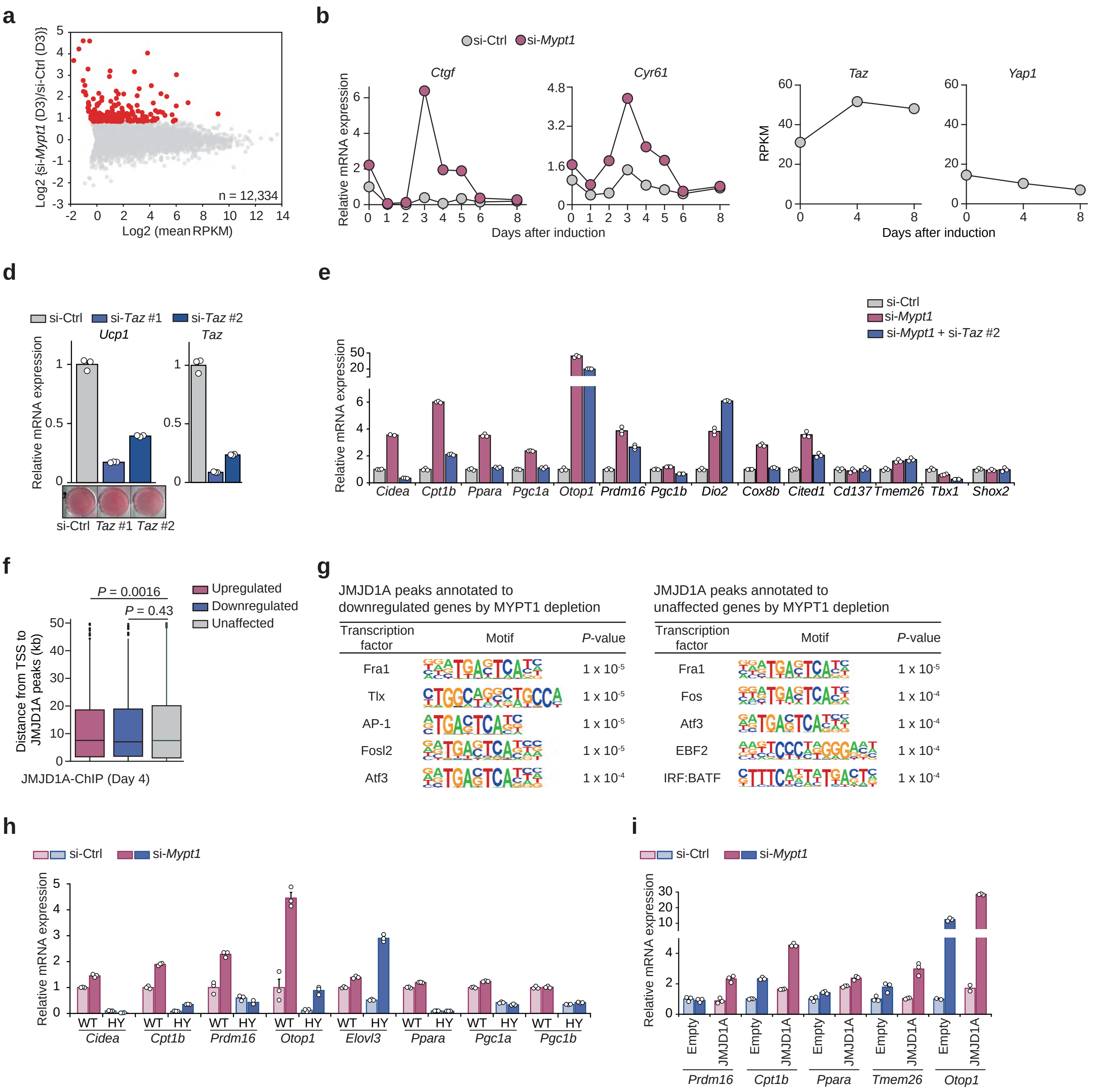
a
Log2 {si-Mypt1 (D3)/si-Ctrl (D3)}
n = 12,334
Log2 (mean RPKM)
b
si-Ctrl
si-Mypt1
Ctgf
Cyr61
Relative mRNA expression
Days after induction
Taz
Yap1
RPKM
Days after induction
d
si-Ctrl
si-Taz #1
si-Taz #2
Ucp1
Taz
Relative mRNA expression
si-Ctrl
Taz #1
Taz #2
e
si-Ctrl
si-Mypt1
si-Mypt1 + si-Taz #2
Relative mRNA expression
Cidea
Cpt1b
Ppara
Pgc1a
Otop1
Prdm16
Pgc1b
Dio2
Cox8b
Cited1
Cd137
Tmem26
Tbx1
Shox2
f
P = 0.0016
P = 0.43
Upregulated
Downregulated
Unaffected
Distance from TSS to JMJD1A peaks (kb)
JMJD1A-ChIP (Day 4)
g
JMJD1A peaks annotated to downregulated genes by MYPT1 depletion
Transcription factor
Motif
P-value
Fra1 1 x 10-5
Tlx 1 x 10-5
AP-1 1 x 10-5
Fosl2 1 x 10-5
Atf3 1 x 10-4
JMJD1A peaks annotated to unaffected genes by MYPT1 depletion
Transcription factor
Motif
P-value
Fra1 1 x 10-5
Fos 1 x 10-4
Atf3 1 x 10-4
EBF2 1 x 10-4
IRF:BATF 1 x 10-4
h
si-Ctrl
si-Mypt1
Relative mRNA expression
WT
HY
Cidea
Cpt1b
Prdm16
Otop1
Elovl3
Ppara
Pgc1a
Pgc1b
i
si-Ctrl
si-Mypt1
Relative mRNA expression
Empty
JMJD1A
Prdm16
Cpt1b
Ppara
Tmem26
Otop1

Supplementary Fig. 3 MYPT1-PP1β depletion induces *Ucp1* by orchestrating epigenetic and direct transcriptional pathways through YAP/TAZ

(**a**) RNA-seq MA plot of 12,334 “expressed” (RPKM > 1) in day 3 im-scWAT cells transfected with si-Ctrl or si-*Mypt1* #2 and differentiated for beige adipocyte. X-axis, log2 mean of RPKM. Y-axis, log2 fold change of RPKM (si-*Mypt1* day 3/si-Ctrl day 3). Red dot represents top 200 genes upregulated by MYPT1 depletion. (**b**) mRNA levels for *Ctgf* (left) and *Cyr61* (right) during beige adipogenesis in im-scWAT cells transfected with si-Ctrl or si-*Mypt1* #2 as determined by qPCR. (**c**) mRNA levels for *Taz* and *Yap1* during beige adipogenesis in im-scWAT cells as determined by RNA-seq analysis and are expressed as RPKM. (**d**) *Ucp1* mRNA levels on day 8 (left) and *Taz* mRNA levels on day 0 (right) were quantified by RT-qPCR in im-scWAT cells transfected with si-Ctrl or si-*Taz*. ORO staining on day 8 (bottom). (**e**) Thermogenic gene expression of im-scWAT transfected with si-Ctrl, si-*Mypt1* #2+si-Ctrl, or si-*Mypt1* #2+si-*Taz* #2 differentiated for beige adipocytes. (**f**) A boxplot showing the distance to TSS of JMJD1A peaks on day 4 annotated to upregulated (fold change > 1.5 and RPKM of si-*Mypt1* transfected cells > 1), downregulated (fold change < 1/1.5 and RPKM of si-Ctrl transfected cells > 1), and unaffected genes by MYPT1 depletion in day 3 im-scWAT cells differentiated for beige adipocytes (n = 945, n = 816, and n = 945 peaks, respectively. The same number of peaks as that annotated to the upregulated genes were randomly extracted from the peaks annotated to unaffected genes to get *P*-value in similar level). The box shows the median and first and third quartiles. *P*-values by two-tailed Mann-Whitney test. (**g**) Motifs identified in JMJD1A peaks on day 4 annotated to downregulated genes or unaffected genes (to get comparable *P*-value, the same numbers of JMJD1A peaks annotated to unaffected genes as that annotated to upregulated genes by MYPT1 depletion were randomly picked) by MYPT1 depletion on day 3 im-scWAT cells differentiated for beige adipogenesis described in **e** by known motif searching. (**h**) Effects of MYPT1-depletion induced thermogenic gene expression on WT and demethylation defective JMJD1A expressing cultured adipocytes. WT- or H1120Y-human JMJD1A-overexpressing im-scWATs were transfected with si-Ctrl or si-*Mypt1* #2, differentiated into beige adipocytes, and harvested on day 8 for qPCR. (**i**) Restoration of thermogenic gene induction by *Mypt1* depletion in catalytically inactive H1120Y-JMJD1A-expressing adipocytes through overexpression of WT-human-JMJD1A. im-scWAT pre-adipocytes expressing JMJD1A-H1120Y that were additionally transduced with either empty or WT-human JMJD1A lentiviral vector were transfected with si-Ctrl or si-*Mypt1* #2 and induced for beige adipogenesis. **b**, **d**, **e**, **h**, **i** Representative of three (**b**) or two (**d**, **e**, **h**, **i**) independent

experiments. Data are mean ± SEM of three technical replicates in **b**, **d**, **e**, **h**, and **i**. Source data are provided as a Source data file.

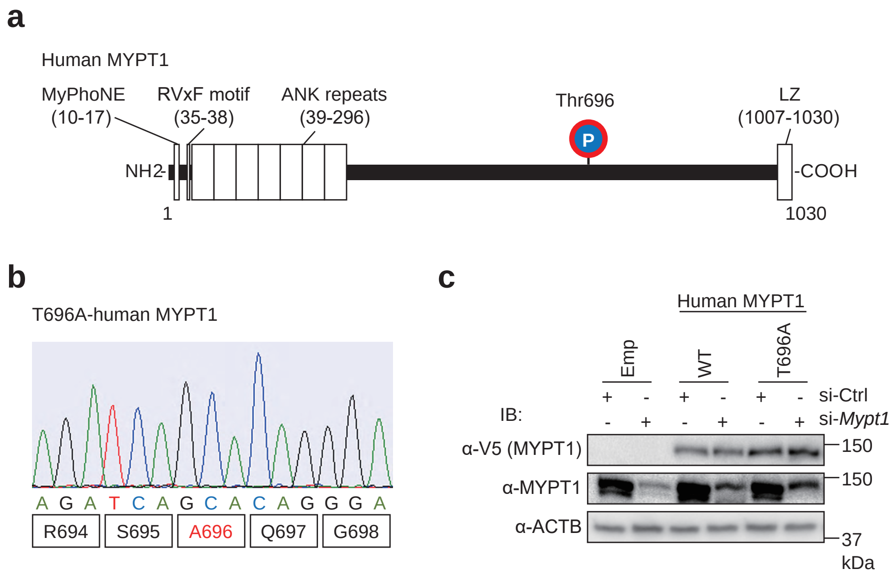
a
Human MYPT1
MyPhoNE (10-17)
RVxF motif (35-38)
ANK repeats (39-296)
Thr696
P
LZ (1007-1030)
NH2-
-COOH
1
1030
b
T696A-human MYPT1
A G A T C A G C A C A G G G A
R694
S695
A696
Q697
G698
c
Human MYPT1
Emp
WT
T696A
IB:
+ - + - + - si-Ctrl
- + - + - + si-Mypt1
α-V5 (MYPT1)
α-MYPT1
α-ACTB
150
150
37
kDa

Supplementary Fig. 4 β-AR-PKA phosphorylation of MYPT1 inhibits MYPT1-PP1β activity

(**a**) Domain structure of human MYPT1. Thr696 phosphorylation site is indicated. MyPhoNE; myosin phosphatase N-terminal element, ANK repeat; ankyrin repeat, LZ; leucine zipper. **(b**) Direct sequencing of retroviral vector for the phosphodefective mutant version of human MYPT1: T696A (Alanine 696: GCA). (**c**) Immunoblotting with anti-V5 (to detect exogenous human MYPT1), anti-MYPT1 (to detect both exogenous [human] and endogenous [murine] MYPT1), or anti-ACTB antibody from WCL of WT- or T696A-human MYPT1-transduced im-scWAT cells on day 0 transfected with si-Ctrl or si-*Mypt1* #3. **c** Representative of two independent experiments. Source data are provided as a Source data file.

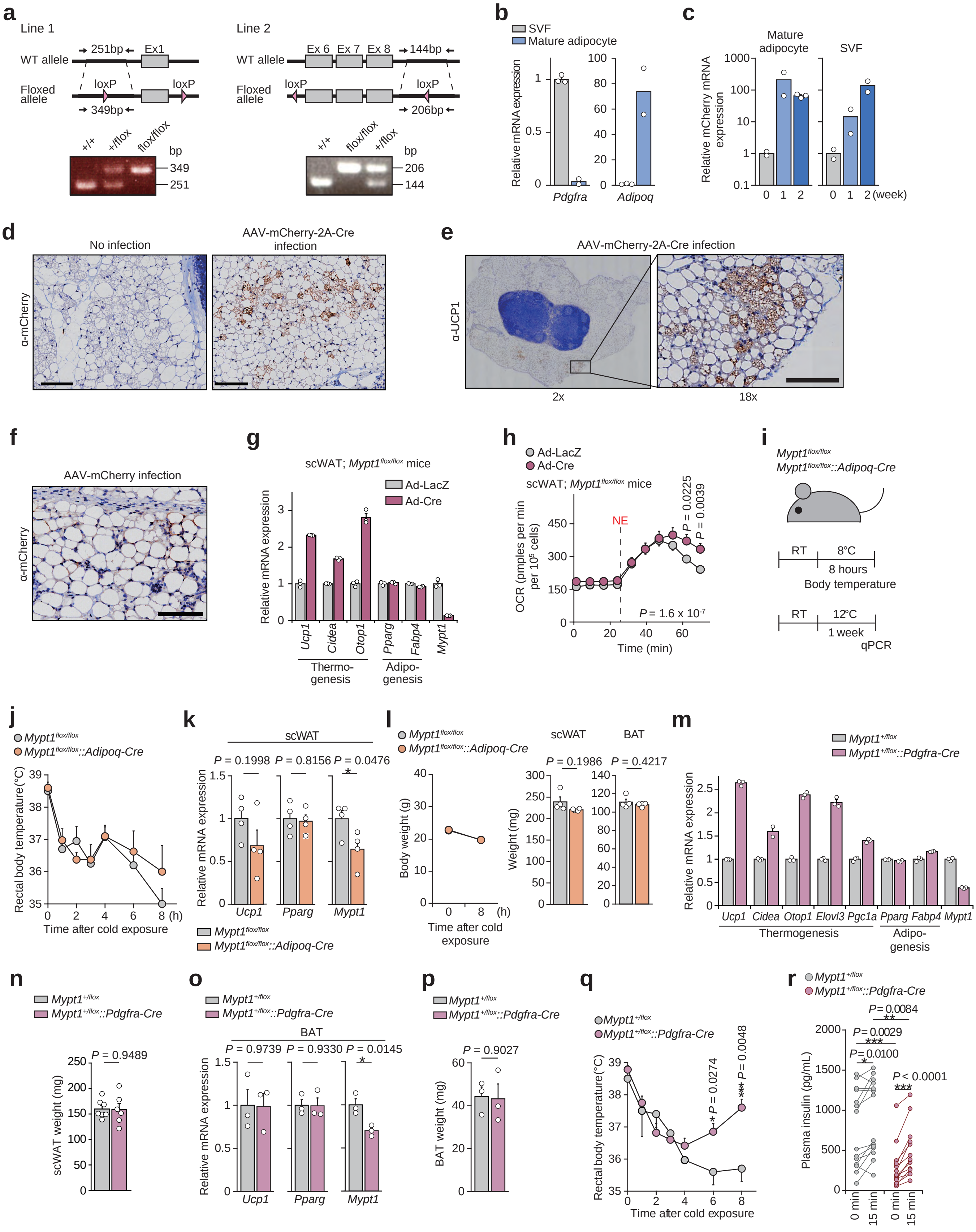
a
Line 1
251bp
Ex1
WT allele
loxP
loxP
Floxed allele
349bp
+/+
+/flox
flox/flox
bp
349
251
Line 2
Ex 6
Ex 7
Ex 8
144bp
WT allele
loxP
loxP
Floxed allele
206bp
+/+
flox/flox
+/flox
bp
206
144
b
SVF
Mature adipocyte
Relative mRNA expression
Pdgfra
Adipoq
c
Mature adipocyte
SVF
Relative mCherry mRNA expression
0 1 2
0 1 2 (week)
d
No infection
AAV-mCherry-2A-Cre infection
α-mCherry
e
AAV-mCherry-2A-Cre infection
α-UCP1
2x
18x
f
AAV-mCherry infection
α-mCherry
g
scWAT; Mypt1 flox/flox mice
Ad-LacZ
Ad-Cre
Relative mRNA expression
Ucp1
Cidea
Otop1
Pparg
Fabp4
Mypt1
Thermo-genesis
Adipo-genesis
h
Ad-LacZ
Ad-Cre
scWAT; Mypt1 flox/flox mice
NE
P = 0.0225
P = 0.0039
P = 1.6 x 10-7
OCR (pmples per min per 10^5 cells)
Time (min)
i
Mypt1 flox/flox
Mypt1 flox/flox::Adipoq-Cre
RT
8°C
8 hours
Body temperature
RT
12°C
1 week
qPCR
j
Mypt1 flox/flox
Mypt1 flox/flox::Adipoq-Cre
Rectal body temperature (°C)
Time after cold exposure
(h)
k
scWAT
P = 0.1998
P = 0.8156
P = 0.0476
Relative mRNA expression
Ucp1
Pparg
Mypt1
Mypt1 flox/flox
Mypt1 flox/flox::Adipoq-Cre
l
Mypt1 flox/flox
Mypt1 flox/flox::Adipoq-Cre
Body weight (g)
Time after cold exposure
scWAT
BAT
P = 0.1986
P = 0.4217
Weight (mg)
m
Mypt1 +/flox
Mypt1 +/flox::Pdgfra-Cre
Relative mRNA expression
Ucp1
Cidea
Otop1
Elovl3
Pgc1a
Pparg
Fabp4
Mypt1
Thermogenesis
Adipo-genesis
n
Mypt1 +/flox
Mypt1 +/flox::Pdgfra-Cre
P = 0.9489
scWAT weight (mg)
o
Mypt1 +/flox
Mypt1 +/flox::Pdgfra-Cre
BAT
P = 0.9739
P = 0.9330
P = 0.0145
Relative mRNA expression
Ucp1
Pparg
Mypt1
p
Mypt1 +/flox
Mypt1 +/flox::Pdgfra-Cre
P = 0.9027
BAT weight (mg)
q
Mypt1 +/flox
Mypt1 +/flox::Pdgfra-Cre
P = 0.0274
P = 0.0048
Rectal body temperature (°C)
Time after cold exposure
r
Mypt1 +/flox
Mypt1 +/flox::Pdgfra-Cre
P = 0.0084
P = 0.0029
P = 0.0100
P < 0.0001
Plasma insulin (pg/mL)
0 min
15 min
0 min
15 min

Supplementary Fig. 5 MYPT1 is crucial for scWAT beiging in mice

(**a**) Schematic representation of MYPT1 knockout strategy and an ethidium bromide-stained agarose gel illustrating PCR products for genotyping WT, *Mypt1*$^{+/flox}$, and *Mypt1*$^{flox/flox}$ mice of lines 1 (left) and 2 (right) (see Methods). Representative of three independent experiments. Ex; Exon. (**b**) *Pdgfra* and *Adipoq* mRNA levels in mature adipocytes and SVFs fraction isolated from AAV8-CMV-mCherry injected scWAT of WT mice. n = 3 for SVF and n = 2 for mature adipocyte. (**c**) *mcherry* mRNA levels in mature adipocytes (left) and SVFs (right) fraction isolated from AAV8-CMV-mCherry injected scWAT of WT mice 0, 1, and 2 weeks after the injection. n = 2 per group except the mature adipocyte samples of 2 weeks (n = 3). (**d**) WT mice without AAV-mCherry-2A-Cre injection and *Mypt1*$^{flox/flox}$ mice (line 2 of *Mypt1* floxed mice, hereafter referred to as line 2) injected with AAV-mCherry-2A-Cre (line 2) were housed at 8°C for 2 weeks and then scWAT was immunostained with mCherry. (**e**) UCP1 immunostaining of scWAT of *Mypt1*$^{flox/flox}$ mice (line 2) injected with AAV-mCherry-2A-Cre and acclimated to 12°C for 1 week. The right panel shows high-magnification images. Scale bar, 100 µm. Tissue samples for UCP1 staining were the same as in Fig. 5d. (**f**) mCherry immunostaining of scWAT of *Mypt1*$^{flox/flox}$ mice (line 2) injected with AAV-mCherry housed at 12°C for 1 week. Scale bar, 100 µm. (**g**) Primary pre-adipocytes isolated from SVF of scWAT of *Mypt1*$^{flox/flox}$ mice (line 1 of *Mypt1* floxed mice, hereafter referred as line 1) were infected with either AxCAN-LacZ (adeno-LacZ) or AxCAN-Cre (Adeno-Cre) which expresses the LacZ and Cre recombinase gene, respectively, under the control of the CAG promoter for 24 h prior to differentiation (day -1 to day 0) and then differentiated for beige adipogenesis. mRNA levels of thermogenic genes, general adipogenic genes, and *Mypt1* on day 8 were quantified by RT-qPCR. (**h**) NE stimulated increase in OCR of primary pre-adipocytes isolated from SVF of scWAT of *Mypt1*$^{flox/flox}$ mice (line 1) infected with adeno-LacZ or -Cre differentiated for beige adipogenesis assessed by Seahorse XF24 extracellular flux analyzer. The dot line indicates the time of the addition for NE. Data are mean ± SEM of 11 (adeno-LacZ) and 10 (adeno-Cre) technical replicates. Two-way repeated measures ANOVA were performed followed by post hoc Student's t-test. (**i**) Schematics of the acute and chronic cold exposure to *Mypt1*$^{flox/flox}$*::Adipoq*-Cre mice. *Mypt1*$^{flox/flox}$ mice were used as controls. (**j**) Cold intolerance in *Mypt1*$^{flox/flox}$*::Adipo*-Cre mice (n = 4 per group) (line 1). Shown is the body temperature of 8-week-old mice at different times after cold exposure at 8°C. (**k**) Expression of *Ucp1*, *Pparg*, and *Mypt1* mRNA in scWAT of *Mypt1*$^{flox/flox}$*::Adipo*-Cre mice (line 1) acclimated to 12°C for 1 week (n = 4 per group). (**l**) Body weight of *Mypt1*$^{flox/flox}$*::Adipo*-Cre mice (line 1) before and after 8 h cold exposure at 8°C (n = 4 per group, left). scWAT and BAT weights of *Mypt1*$^{flox/flox}$*::Adipo*-Cre mice acclimated to 12°C for 1 week (n = 4 per group, right). (**m**) Thermogenic genes, general adipogenic genes, and *Mypt1* mRNA levels were quantified by RT-qPCR at day 8 in primary pre-adipocytes isolated from SVF of scWAT of *Mypt1*$^{+/flox}$*::Pdgfra*-Cre mice (line 1) differentiated for beige adipogenesis. (**n**) The weights of scWAT of *Mypt1*$^{+/flox}$*::Pdgfra*-Cre mice (line 2) housed at RT Data are mean ± SEM (*Mypt1*$^{+/flox}$ n =

7; *Mypt1*$^{+/flox}$*::Pdgfra*-Cre n = 6)). (**o, p**) Expression of *Ucp1*, *Pparg*, and *Mypt1* mRNA in BAT of *Mypt1*$^{+/flox}$*::Pdgfra*-Cre mice (line 2) housed at RT (**o**). The weights of BAT of *Mypt1*$^{+/flox}$*::Pdgfra*-Cre mice (line 2) housed at RT (**p**). n = 3 per group. (**q**) Cold intolerance in *Mypt1*$^{+/flox}$*::Pdgfra*-Cre mice (line 2). Data are mean ± SEM (*Mypt1*$^{+/flox}$ n = 3; *Mypt1*$^{+/flox}$*::Pdgfra*-Cre n = 6). Shown is the body temperature at different times after cold exposure at 8°C. (**r**) Glucose stimulated insulin secretion (GSIS) in each genotype group mice fed on HFD in Fig. 5j (*Mypt1*$^{+/flox}$: n = 14; *Mypt1*$^{+/flox}$*::Pdgfra*-Cre: n = 14) (line 2). The data at 0 min were the same as those in Fig. 5l. Paired two-tailed t-test for comparison of glucose effect. Unpaired two-tailed Student's t-test for comparison of groups. **g** and **m** representative of two independent experiments. Data are mean ± SEM of three technical replicates in **g** and **m**. **k**, **l**, **n**, **o**, **p**, and **q** Unpaired two-tailed Student's t-test (log10-tansformed data are used in **o** to conform to normality). Source data are provided as a Source data file.

Supplementary References

1. Abe, Y. *et al.* JMJD1A is a signal-sensing scaffold that regulates acute chromatin dynamics via SWI/SNF association for thermogenesis. *Nat Commun* **6**, 7052 (2015).
2. Heinz, S. *et al.* Simple combinations of lineage-determining transcription factors prime cis-regulatory elements required for macrophage and B cell identities. *Mol Cell* **38**, 576-589 (2010).
3. Langmead, B. & Salzberg, S.L. Fast gapped-read alignment with Bowtie 2. *Nat Methods* **9**, 357-359 (2012).
4. Bolger, A.M., Lohse, M. & Usadel, B. Trimmomatic: a flexible trimmer for Illumina sequence data. *Bioinformatics* **30**, 2114-2120 (2014).
5. Li, H. *et al.* The Sequence Alignment/Map format and SAMtools. *Bioinformatics* **25**, 2078-2079 (2009).
6. Quinlan, A.R. & Hall, I.M. BEDTools: a flexible suite of utilities for comparing genomic features. *Bioinformatics* **26**, 841-842 (2010).
7. Dobin, A. *et al.* STAR: ultrafast universal RNA-seq aligner. *Bioinformatics* **29**, 15-21 (2013).
8. Feng, J. *et al.* GFOLD: a generalized fold change for ranking differentially expressed genes from RNA-seq data. *Bioinformatics* **28**, 2782-2788 (2012).
9. Chen, S., Zhou, Y., Chen, Y. & Gu, J. fastp: an ultra-fast all-in-one FASTQ preprocessor. *Bioinformatics* **34**, i884-i890 (2018).
10. Ramírez, F. *et al.* deepTools2: a next generation web server for deep-sequencing data analysis. *Nucleic Acids Res* **44**, W160-165 (2016).
