# Supplementary material for: MYPT1-PP1β phosphatase negatively regulates both chromatin landscape and co-activator recruitment for beige adipogenesis
Source: Nat Commun. 2022 Sep 29;13:5715. doi: 10.1038/s41467-022-33363-0 (PMC9523048; doi:10.1038/s41467-022-33363-0)
Supplement: Supplementary file 3 — Description of Additional Supplementary Files [file 41467_2022_33363_MOESM3_ESM.pdf]

Description of Additional Supplementary Files

File Name: Supplementary Data 1
Description: List of identified proteins by proteomics.
