# Supplementary material for: MYPT1-PP1β phosphatase negatively regulates both chromatin landscape and co-activator recruitment for beige adipogenesis
Source: Nat Commun. 2022 Sep 29;13:5715. doi: 10.1038/s41467-022-33363-0 (PMC9523048; doi:10.1038/s41467-022-33363-0)
Supplement: Supplementary file 5 — Reporting Summary [file 41467_2022_33363_MOESM5_ESM.pdf]

nature portfolio

Corresponding author(s): Juro Sakai

Last updated by author(s): 2022/9/6

Reporting Summary

Nature Portfolio wishes to improve the reproducibility of the work that we publish. This form provides structure for consistency and transparency in reporting. For further information on Nature Portfolio policies, see our Editorial Policies and the Editorial Policy Checklist.

Statistics

For all statistical analyses, confirm that the following items are present in the figure legend, table legend, main text, or Methods section.

n/a	Confirmed	
☐	☒	The exact sample size (*n*) for each experimental group/condition, given as a discrete number and unit of measurement
☐	☒	A statement on whether measurements were taken from distinct samples or whether the same sample was measured repeatedly
☐	☒	The statistical test(s) used AND whether they are one- or two-sided<br>*Only common tests should be described solely by name; describe more complex techniques in the Methods section.*
☒	☐	A description of all covariates tested
☒	☐	A description of any assumptions or corrections, such as tests of normality and adjustment for multiple comparisons
☐	☒	A full description of the statistical parameters including central tendency (e.g. means) or other basic estimates (e.g. regression coefficient) AND variation (e.g. standard deviation) or associated estimates of uncertainty (e.g. confidence intervals)
☐	☒	For null hypothesis testing, the test statistic (e.g. *F*, *t*, *r*) with confidence intervals, effect sizes, degrees of freedom and *P* value noted<br>*Give P values as exact values whenever suitable.*
☒	☐	For Bayesian analysis, information on the choice of priors and Markov chain Monte Carlo settings
☒	☐	For hierarchical and complex designs, identification of the appropriate level for tests and full reporting of outcomes
☐	☒	Estimates of effect sizes (e.g. Cohen's *d*, Pearson's *r*), indicating how they were calculated

Our web collection on statistics for biologists contains articles on many of the points above.

Software and code

Policy information about availability of computer code

Data collection	Illumina Sequencing Control Software v2.10.17 and HiSeq Control Software v2.2.58 for ChIP-seq and RNA-seq. Xcalibur2.2 for proteomics.
Data analysis	Trimmomatic0.39, Bowtie2.2.4.2, Samtools1.12, Picard2.25.5, Deeptools3.5.1, Enrichr, Homer4.11, STAR2.7.9a, GFOLD1.14, FASTP0.20.1 for ChIP-seq and RNA-seq analyses. ProteoWizard v3.0.11018, ProteoDiscoverer v1.3.0.339 and v2.1, MaxQuant v1.6.2.10, Mascot v2.4 and v2.6.1 for proteomics. Scansite 4.1.0 for database search for identification of kinase consensus sequences. ImageJ1.53k for quantification of immunoblot.

For manuscripts utilizing custom algorithms or software that are central to the research but not yet described in published literature, software must be made available to editors and reviewers. We strongly encourage code deposition in a community repository (e.g. GitHub). See the Nature Portfolio guidelines for submitting code & software for further information.

Data

Policy information about availability of data

All manuscripts must include a data availability statement. This statement should provide the following information, where applicable:

- Accession codes, unique identifiers, or web links for publicly available datasets
- A description of any restrictions on data availability
- For clinical datasets or third party data, please ensure that the statement adheres to our policy

Data supporting this study are available from the corresponding authors upon reasonable request. RNA-seq transcriptome data and JMJD1A ChIP-seq data (day 0 and day 4) were deposited in the Gene Expression Omnibus (GEO) database with accession number GSE202506 (https://www.ncbi.nlm.nih.gov/geo/query/acc.cgi?acc=GSE202506). JMJD1A ChIP-seq data (day 8) has already been published and deposited in the GEO database GSE107901 (https://www.ncbi.nlm.nih.gov/geo/

query/acc.cgi?acc=GSE107901). Proteomic data were deposited in ProteomeX change Consortium under the accession number PXD031210, PXD031896, and PXD031897. The Mus SwissProt database (v. 2017_04, 2019_04, and 2021_04) was used for proteome analyses. Source data are provided with this paper.

Field-specific reporting

Please select the one below that is the best fit for your research. If you are not sure, read the appropriate sections before making your selection.

☒ Life sciences ☐ Behavioural & social sciences ☐ Ecological, evolutionary & environmental sciences

For a reference copy of the document with all sections, see nature.com/documents/nr-reporting-summary-flat.pdf

Life sciences study design

All studies must disclose on these points even when the disclosure is negative.

Sample size	Sample sizes were selected based on previously studies with similar type of experiments (PMID:25948511, 26590716, 29674659).
Data exclusions	No data were excluded.
Replication	Experiments were repeated at least twice with the independent biological samples using similar experimental conditions or otherwise mentioned in the respective figure legends, main text or methods section.
Randomization	All experiments were conducted using randomly assigned animals and cells culture populations
Blinding	For all experiments using cells and mice, blinding was not relevant because cells and mice had to be genotyped by PCR.

Reporting for specific materials, systems and methods

We require information from authors about some types of materials, experimental systems and methods used in many studies. Here, indicate whether each material, system or method listed is relevant to your study. If you are not sure if a list item applies to your research, read the appropriate section before selecting a response.

Materials & experimental systems

n/a	Involved in the study	
☐	☒	Antibodies
☐	☒	Eukaryotic cell lines
☒	☐	Palaeontology and archaeology
☐	☒	Animals and other organisms
☒	☐	Human research participants
☒	☐	Clinical data
☒	☐	Dual use research of concern

Methods

n/a	Involved in the study	
☐	☒	ChIP-seq
☒	☐	Flow cytometry
☒	☐	MRI-based neuroimaging

Antibodies

Antibodies used

Following are the antibodies used in this study:
1. Anti-mouse JMJD1A mouse mAb IgG-F0618 (RCAST, The University of Tokyo, Japan)
2. Anti-mouse JMJD1A mouse mAb IgG-F0231 (RCAST, The University of Tokyo, Japan)
3. Anti-human JMJD1A mouse mAb IgG-F3640 (RCAST, The University of Tokyo, Japan)
4. Anti-mouse P-JMJD1A (pS265) rabbit pAb 11890-2 (RCAST, The University of Tokyo, Japan)
5. Anti-RLC rabbit mAb D18E2 (Cell Signaling Technology, 8505)
6. Anti-P-RLC (pSer19) rabbit pAb (Cell Signaling Technology, 3671)
7. Anti-MYPT1 rabbit pAb (Cell Signaling Technology, 2634)
8. Anti-P-MYPT1 (pThr696) rabbit pAb (Sigma-Aldrich, ABS45)
9. Anti-ACTB mouse mAb AC-15 (Sigma-Aldrich, A5441)
10. Anti-V5 mouse mAb (Thermo Scientific, R960-25)
11. Anti-H3K9me2 mouse mAb IgG-6D11 (Institute of Innovative Research, Tokyo Institute of Technology, Japan)
12. Anti-UCP1 rabbit pAb (Abcam, ab23841)
13. Anti-mCherry mouse mAb 1C51 (Abcam, ab125096)
14. Anti-UCP1 mouse mAb 536435 (R&D Systems, MAB6158)
15. Anti-TOM20 rabbit pAb (Proteintech, 11802-1-AP)
16. Anti-FLAG mouse mAb M2 (Sigma-Aldrich, F3165)
17. Anti-mouse IgG-HRP (Sigma-Aldrich, A4416)
18. Anti-rabbit IgG-HRP (Sigma-Aldrich, A0545)

March 2021

Validation

The validation of antibodies used was performed by the individual companies and publications.
1. Anti-mouse JMJD1A mouse mAb IgG-F0618: Specificity was validated by immunoblot (Abe Y. et al., Nat Commun., 2015, 6, 7052, PMID: 25948511)
2. Anti-mouse JMJD1A mouse mAb IgG-F0231: Specificity was validated by immunoblot (Abe Y. et al., Nat Commun., 2015, 6, 7052, PMID: 25948511)
3. Anti-human JMJD1A mouse mAb IgG-F3640: Specificity was validated by immunoblot (Abe Y. et al., Nat Commun., 2015, 6, 7052, PMID: 25948511)
4. Anti-mouse P-JMJD1A (pS265) rabbit pAb 11890-2: Specificity was validated by immunoblot (Abe Y. et al., Nat Commun., 2015, 6, 7052, PMID: 25948511)
5. Anti-RLC rabbit mAb D18E2 - Manufacturer's statement: ' Monoclonal antibody is produced by immunizing animals with a synthetic peptide corresponding to residues near the carboxy terminus of human myosin light chain 2 protein. Applications: Western Blot. Species reactivity: Human, Mouse, Rat.' (https://www.cellsignal.jp/products/primary-antibodies/myosin-light-chain-2-d18e2-rabbit-mab/8505)
6. Anti-P-RLC (pSer19) rabbit pAb - Manufacturer's statement: ' Polyclonal antibodies are produced by immunizing animals with a synthetic phosphopeptide corresponding to residues surrounding Ser19 of human myosin light chain 2. Applications: Western Blot, Immunocytochemistry. Species reactivity: Human, Mouse, Rat, D. melanogaster.' (https://www.cellsignal.jp/products/primary-antibodies/phospho-myosin-light-chain-2-ser19-antibody/3671)
7. Anti-MYPT1 rabbit pAb - Manufacturer's statement: ' Polyclonal antibodies are produced by immunizing animals with a synthetic peptide correspondent to amino-terminal residues of human MYPT1. Applications: Western Blot. Species reactivity: Human, Mouse, Rat, Hamster, Monkey.' (https://www.cellsignal.jp/products/primary-antibodies/mypt1-antibody/2634)
8. Anti-P-MYPT1 (pThr696) rabbit pAb - Manufacturer's statement: 'This antibody recognizes phosphorylated MYPT1 at Thr696. Applications: Western Blot. Species reactivity: Human, Mouse, Primate, Rat, Canine, Xenopus, Horse, Chicken.' (https://www.merckmillipore.com/JP/ja/product/Anti-phospho-MYPT1-Thr696-Antibody,MM_NF-ABS45)
9. Anti-ACTB mouse mAb AC-15 -Manufacturer's statement: 'Anti-β-Actin antibody, Mouse Monoclonal 1, 2 recognizes an epitope located on the N-terminal end of the β-isoform of actin. Monoclonal mouse anti-actin was used as a loading control for western blot analysis of rat liver protein lysates. The antibody has also been used for western blot at 0.5-1 μg/mL using cell extract of human foreskin fibroblasts or chicken fibroblasts. Species reactivity: pig, Hirudo medicinalis, bovine, rat, canine, feline, human, rabbit, carp, mouse, guinea pig, chicken, sheep.' (https://www.sigmaaldrich.com/JP/ja/product/sigma/a5441)
10. Anti-V5 mouse mAb - Manufacturer's statement: 'R960-25 recognizes amino acid sequence: -Gly-Lys-Pro-Ile-Pro-Asn-Pro-Leu-Leu-Gly-Leu-Asp-Ser-Thr-. This antibody is functionally tested against 20 ng of an E. coli expressed fusion protein containing a V5 epitope using a chemiluminescent substrate at a 1 minute exposure. Applications; WB, IHC, Flow cytometry, ELISA, IP, ChIP.' (https://www.thermofisher.com/antibody/product/V5-Tag-Antibody-Monoclonal/R960-25)
11. Anti-H3K9me2 mouse mAb IgG-6D11: Specificity was validated by ELISA, ChIP, WB, and IF (Kimura H. et al., Cell Struct. Funct., 2008, 33, 61-73, PMID: 18227620)
12. Anti-UCP1 rabbit pAb - Manufacturer's statement: ' Synthetic peptide corresponding to Human UCP1 aa 100-200 conjugated to keyhole limpet haemocyanin. Applications: Western Blot. Species reactivity: Mouse, Rat.' (https://www.abcam.co.jp/ucp1-antibody-ab23841.html)
13. Anti-mCherry mouse mAb 1C51 - Manufacturer's statement: 'Immunogen: Recombinant full length protein corresponding to mCherry. Applications: WB, ICC/IF, IHC-P.' (https://www.abcam.co.jp/mcherry-antibody-1c51-ab125096.html)
14. Anti-UCP1 mouse mAb 536435 - Manufacturer's statement: 'Immunogen: E. coli-derived recombinant human UCP1 Met1-Thr307. Applications: Western Blot, Simple Western, Immunocytochemistry, Intracellular Staining by Flow Cytometry. Species reactivity: Human, Mouse.' (https://www.rndsystems.com/products/human-mouse-ucp1-antibody-536435_mab6158#product-details)
15. Anti-TOM20 rabbit pAb - Manufacturer's statement: 'Immunogen: TOM20 fusion protein Ag2378. Applications: Western Blot, Immunoprecipitation, Immunohistochemistry, Immunofluorescence. Species reactivity: Human, Mouse, Rat.' (https://www.ptglab.co.jp/products/TOM20-Antibody-11802-1-AP.htm)
16. Anti-FLAG mouse mAb M2 -Manufacturer's statement: 'Anti Flag M2 antibody is used for the detection of Flag fusion proteins. Application: immunoblotting, immunoprecipitation, immunocytochemistry, immunofluorescence, ELISA, EIA, chromatin immunoprecipitation, electron microscopy, flow cytometry, supershift assays.' (https://www.sigmaaldrich.com/US/en/product/sigma/f3165)
17. Anti-mouse IgG-HRP -Manufacturer's statement: 'Anti-Mouse IgG (whole molecule)-Peroxidase antibody is suitable for use in immunoblot. Species reactivity: mouse.' (https://www.sigmaaldrich.com/US/en/product/sigma/a4416)
18. Anti-rabbit IgG-HRP -Manufacturer's statement: 'Anti-Rabbit IgG (whole molecule)–Peroxidase antibody has been used in western blotting, immunofluorescence staining, immunochemistry and immunoprecipitations. Species reactivity: rabbit.' (https://www.sigmaaldrich.com/US/en/product/sigma/a0545)

Eukaryotic cell lines

Policy information about cell lines

Cell line source(s)

3T3-L1 (ATCC); AAV293 (Agilent); Plat-E (Cosmo Bio Co. Ltd.); NIH-3T3 (ATCC). Pre-adipocytes were isolated from the inguinal WAT of either wild type or Mypt1 flox/flox mice and immortalized by infecting retrovirus expressing SV-Large T antigen to obtain im-scWAT cells.

Authentication

3T3-L1 and im-scWAT cells were validated by proteomics, ChIP-seq, and RNA-seq as mouse cell line (i.e. mouse-unique sequences detected). AAV293, NIH3T3, and Plat-E cells were not authenticated.

Mycoplasma contamination

Four cell lines are tested for mycoplasma contamination and found to be negative.

Commonly misidentified lines (See ICLAC register)	No misidentified cell lines were used in this study.

Animals and other organisms

Policy information about studies involving animals; ARRIVE guidelines recommended for reporting animal research

Laboratory animals	Mypt1 floxed mice and Adipoq-Cre mice were generated as described in Methods. Pdgfrα-Cre (stock 013148) mice were purchased from Jackson Laboratories. Male or female Mypt1 flox/flox mice aged 18-34 weeks, male or female Mypt1 +/flox::Pdgfra-Cre mice aged 5-33, and male Mypt1 flox/flox::Adipoq-Cre mice aged 8 weeks were used for experiments. Littermate controls with same sex were used for experiments. Mice were maintained in a temperature- and humidity-controlled environment under a 12h light/12h dark cycle (08:00-20:00) at constant temperature (23°C) with free access to food and water. Animals were fed a normal chow diet (CE-2, CLEA Japan Inc.) from the age of 4 weeks or HFD, consisting of 58.0% fat, 15.0% protein, and 27.0% carbohydrate, from the age of 5 to 7 weeks
Wild animals	No wild animals were used in this study.
Field-collected samples	No field collected samples were used.
Ethics oversight	All animal studies were approved by the Animal Care and Use Committee of The University of Tokyo and Tohoku University.

Note that full information on the approval of the study protocol must also be provided in the manuscript.

ChIP-seq

Data deposition

☒ Confirm that both raw and final processed data have been deposited in a public database such as GEO.

☒ Confirm that you have deposited or provided access to graph files (e.g. BED files) for the called peaks.

Data access links *May remain private before publication.*	ChIP-seq data were deposited in the Gene Expression Omnibus (GEO) database with accession numbers GSE202506.<br>https://www.ncbi.nlm.nih.gov/geo/query/acc.cgi?acc=GSE202506<br>The link is private until publication of the manuscript.
Files in database submission	FASTQ and bigwig files were deposited.
Genome browser session (e.g. UCSC)	Reviewer access links of Genome browser session are as follow:<br>https://genome-asia.ucsc.edu/s/Yoshihiro%20Matsumura/mm9_Ucp1

Methodology

Replicates	JMJD1A ChIP-seq was done in one replicate.
Sequencing depth	Number of total reads in each sample:<br>im-scWAT_D0_JMJD1A: 29,235,913<br>im-scWAT_D4_JMJD1A: 28,407,382
Antibodies	1. Anti-mouse JMJD1A mouse mAb IgG-F0618 (Tohoku University Graduate School of Medicine or Division of Metabolic Medicine, Japan) (RCAST, The University of Tokyo, Japan)<br>2. Anti-mouse JMJD1A mouse mAb IgG-F0231 (Tohoku University Graduate School of Medicine or Division of Metabolic Medicine, Japan) (RCAST, The University of Tokyo, Japan)
Peak calling parameters	JMJD1A: Homer (-style histone for broad peak)
Data quality	Number of peaks in each sample using above peak calling parameter:<br>im-scWAT_D0_JMJD1A: 36,023<br>im-scWAT_D4_JMJD1A: 46,585
Software	Trimmomatic0.39; Bowtie2.2.4.2; Samtools1.12; Deeptools3.5.1; Homer4.11.
